# What animals do not do or fail to find: A novel observational approach for studying cognition in the wild

**DOI:** 10.1002/evan.21794

**Published:** 2019-08-16

**Authors:** Karline R. L. Janmaat

**Affiliations:** ^1^ Max Planck Institute for Evolutionary Anthropology Leipzig Germany; ^2^ Institute for Biodiversity and Ecosystem Dynamics University of Amsterdam Amsterdam The Netherlands

**Keywords:** animal cognition, brain evolution, chimpanzees, field‐based studies, foraging behavior, fruit, observational approach, rainforest

## Abstract

To understand how our brain evolved and what it is for, we are in urgent need of knowledge about the cognitive skills of a large variety of animal species and individuals, and their relationships to rapidly disappearing social and ecological conditions. But how do we obtain this knowledge? Studying cognition in the wild is a challenge. Field researchers (and their study subjects) face many factors that can easily interfere with their variables of interest. Although field studies of cognition present unique challenges, they are still invaluable for understanding the evolutionary drivers of cognition. In this review, I discuss the advantages and urgency of field‐based studies on animal cognition and introduce a novel observational approach for field research that is guided by three questions: (a) *what do animals fail to find?*, (b) *what do they not do?*, and (c) *what do they only do when certain conditions are met?* My goal is to provide guidance to future field researchers examining primate cognition.

## 1 THE COMPARATIVE APPROACH

Comparison is fundamental in understanding the evolution of cognition (Box 1). Over the past decades, scientists from the fields of anthropology, psychology, and biology have employed the comparative (phylogenetic) method to gain insights into the evolution of the animal mind^1^, ^2^, ^3^, ^4^ and to identify cognitive traits that are unique to humans and those that are shared with other animals. This work has focused on a variety of topics, ranging from comparisons of primate skulls^2^, ^4^, ^5^ with that of gene‐regulatory networks driving the earliest stages of cortical development.^6^ Additional research on the evolution of cognitive traits is conducted by inferring cognitive abilities from observed behaviors across species.^7^, ^8^ By linking differences in cognitive abilities with differences in current socio‐ecological circumstances, hypotheses about the evolutionary pressures that contributed to the positive selection of these abilities can be tested and this can provide answers to the question of why the traits evolved.^3^, ^4^, ^9^ Drawing inference about cognitive abilities from behavior is, however, not straightforward. Behavioral scientists have therefore developed two approaches: the experimental approach and the observational approach.

Box 1 **: Cognition**

Cognition, defined as the mechanisms by which animals acquire, process, store, and act on information from the environment,^8^ can result in declarative knowledge (knowing that) and procedural knowledge (knowing what to do^8^). For example, when an animal is searching for food, it could have knowledge about the exact locations of, and directions between, the food and a small hill, or it could simply only know that if it wants to find food, it needs to turn right when it reaches the small hill. Knowing what an animal knows and what cognitive mechanisms it uses is not simply derived from observing what an animal does. Hence, behavioral science has developed experimental and observational approaches to infer cognition from behavior. Different types of cognition can lead to a variety of knowledge that can help an animal to find, access, and guard food and mates. For example, to find food, animals may use foraging cognition,^33^ that is, mechanisms that acquire, process, store, and act upon (a) sensory information about the cues emitted by foods,^69^, ^91^, ^119^, ^183^ (b) spatial information of the locations and efficient route of travel,^18^, ^155^, ^184^ (c) temporal information of the timing of a visit, or return,^58^, ^176^, ^184^ (d) ecological information of the characteristics of food sources and competitors (e.g., level of ephemerality, synchrony, fruit production, and depletion rates^19^, ^70^, ^71^, ^74^, ^186^, ^187^), and (e) social information about the decisions or knowledge of group members.^33^, ^188^, ^189^ All these information types can either result in declarative or procedural knowledge. A cognitive scientist's challenge is to find out by looking at the outside what type of knowledge is emerging on the inside.

## 2 HOW TO STUDY ANIMAL COGNITION?

The first approach to infer cognitive mechanisms from behavior, often seen as the “gold standard” in cognitive science, is the experimental approach. Shettleworth states, “It is almost never possible to tell without experimental analysis what kinds of processes are reflected in a given behavior”^8^ (p. 5). In lab or field‐based experiments, scientists manipulate predictor variables that are thought to influence the animal's behavior. For example, by placing an animal into a new environment and minimizing the number of landmarks it is familiar with, we can test the animal's ability to make novel shortcuts between newly learned food locations. This helps us to infer what type of mental representation of space the animal made.^10^ The second approach is to observe behavior in a natural environment without manipulation (hereafter: an observational approach). In this particular example, we would wait until an animal disperses into novel areas to know whether it can find novel shortcuts or not. Clearly, experiments can make research more time‐efficient and at the same time make it easier to distinguish between cause and effect. By comparing manipulated with unmanipulated control conditions, we can infer that it was only the manipulated variable that affected the animal's response and not any other variable that happened to change simultaneously with the variable of interest. This is particularly important when such variables are naturally associated with each other. For example, if we play back an alarm call and the targeted monkey reacts, we can infer that it is only the sound that it reacted to and not the smell or the body language of the animal that emitted the call.^11^ In other words, when multiple sensory stimuli are always experienced simultaneously by the receiver in a natural situation, an experiment is the only way to infer what information the receiver acts on.

Clearly, we have to assume that causal relations are present,^12^ yet it is important to keep in mind that causality can never be proven, whatever approach we take.^12^, ^13^ Experiments simply make it more likely that a change in one variable leads to a change in another variable, *ceteris paribus* (other things being equal). Since other things rarely are equal, a balanced experimental design is required. The difference between experimental and control conditions, such as the vegetation density during a playback of an alarm call in a tropical forest,^11^ may not always be easy to measure. To better account for such a confounding variables (e.g., a vegetation type that facilitates a predator's attack), we should balance the order and number of control and experimental trials. In addition, we should randomize the assignment of individuals to trials, to account for a difference between individuals. However, this can be difficult in most field and zoo experiments, where individuals cannot be separated from the group. In addition, differences between experimental and control trials are less likely to be balanced out when the number of trials one can conduct is limited due to habituation effects or a small number of available subjects.

Differences between experimental and observational approaches are not always as clear as is generally assumed. All experiments require observation, and experiments may not control all possible confounding variables. In these cases, experimental studies can, by a posteriori means, statistically control some confounding factors, such as motivation, that could not be controlled by the experimental design.^14^, ^15^, ^16^, ^17^ Similarly, observational studies can, by a priori or a posteriori means, control confounding variables, which increases the likelihood of finding a cause and effect relationships substantially.^18^, ^19^, ^20^ In fact, some experimental scientists describe experiments and nonexperimental observational studies as not categorically distinct methods, but rather place them at two ends of a continuum of planned versus post hoc control for variation of predictor variables (see^21^ for further details).

In addition to being part of a continuum, both approaches are inseparable. The list of experimental studies in cognitive science that were initially inspired by observational studies on foraging, predator avoidance, and social behavior, is extensive.^14^, ^22^, ^23^, ^24^, ^25^, ^26^, ^27^, ^28^ Yet, more importantly, experiments have little value without previous field‐based observational work. To make sense of an animal's reaction to a manipulation, we first need to know how it reacts to naturally occurring variation in that same variable. Furthermore, knowledge about failures in experimental design^29^ (e.g., due to distortions in broadcast speaker sound) only become apparent once we know that the reaction to the manipulated variable is different from the animal's reaction to naturally occurring variation in that same variable. Similarly, experiments preceded by observational recordings of the subjects' behavior can help to explain cognitive performance. Such combined approaches can, for example, help researchers avoid selecting individuals that were recently involved in a social conflict before joining a cooperative experimental task.^30^

## 3 WHERE TO STUDY COGNITION?

It is not always considered ethically justifiable to manipulate wild and protected animals. Therefore, many experiments that focus on highly endangered and protected animals, such as great apes, take place in laboratories or zoos, where experimental manipulation does not distract from or delay animals from finding natural foods or detecting predators.^8^ However, that is often not the first argument brought forward to support this choice of research location. When discussing the pros and cons of field‐based versus captive‐based studies in primatology, Tomasello & Call^31^ wrote that “Other methodological challenges for field approaches to primate cognition emanate from the impossibility of controlling all relevant factors under ‘wild’ conditions.”

For example, if we refer back to the observation of the animal that dispersed to a new area, we will never know whether the animal's ability to make new shortcuts in the novel area resulted from it using its own cognitive abilities, or by it simply following the new group members it encountered.^32^ This leads us to the question of where one can best study animal cognition.^33^, ^34^ Debates on what is the best environment to do so have been numerous.^23^, ^37^, ^38^ In some fields of science, these debates led to the realization that a collaboration between lab and field‐based science, also termed the *synthetic* approach, is essential for improving scientific insights.^9^, ^39^, ^40^, ^41^, ^42^

In the field of primate cognition, which is most prominent in investigations on the origins of human cognition, the debates seem to have led to an alienation of each other's work. In fact, field‐based and captive‐based primatologists rarely read or cite each other's work^33^, ^34^, ^35^, ^36^ (Box 2). This situation is unfortunate, because it is especially the comparison of natural habitat and captive studies can inform us about evolution.^40^, ^41^

Box 2 **The Imbalanced Distribution and Diffusion of Knowledge in Primate Cognition**

A recent study investigated how knowledge derived from research in either captive or natural environments is represented in the literature on primate cognition, and to what degree captive and field approaches for data collection are used in these two types of studies.^190^ For this study, Glabischnig^190^ selected 16 review and theoretical papers focusing on primate cognition and the types of studies (field vs. captive) that were cited by the respective authors were counted (Tables S1 and S2).

3.1 **Distribution of study types across all cited publications**

Glabischnig^190^ found 583 (66.55%) references to studies conducted on primates in captive environments and 293 (33.45%) references to studies in natural environments (Tables S1 and S2). These figures suggest a highly unbalanced availability or distribution of knowledge on primate cognition from natural versus captive environments. Captive‐based studies largely applied experimental techniques and only 15% used purely observational methods in their research. In contrast, studies in natural environments mainly applied observational methods and used experimental techniques in 28% of cases (rates include studies that incorporated both experiment and observation [Table S1]).

Captive‐based studies were cited at a higher rate than field‐based studies by captive‐oriented primatologists (317 captive vs. 48 natural studies cited). Citations of their field‐based colleagues showed a more equal distribution (266 captive vs. 240 natural studies cited). This was also reflected by the average ratio of captive‐based/field‐based studies for captive‐oriented (8.27) and field‐oriented primatologists (1.28; Table S2). In addition, there is a notable difference in the kind of field‐based studies that were referenced by the two different types of researchers. While field‐oriented researchers cited experimental studies in the wild more than 30% of cases, their captive‐oriented colleagues cited the same type of study about 8% of the time (percentages include also studies that include field experiments as well as a combination of experimental and observation studies [Table S3]).

There was one outlier among the publications from field‐oriented primatologists with a high lab/field ratio (ratio: 4.59; Table S2). Interestingly, this publication is a collaborative paper between field‐oriented and captive‐oriented researchers. The collaboration seems to have resulted in a lower ratio of lab/field studies than the average ratio for captive‐oriented primatologists, as well as a much higher absolute count of field study citations than any paper from captive‐oriented primatologists (Table S2).

A classic selection study in the field of evolutionary biology provides perhaps the best example of a comparative field‐based and lab‐based study that led to new insights. In this study, a set of lab experiments found that guppies (*Poecilia reticulata*) derived from high predation localities had delayed senescence in comparison to counterparts from low predation localities, while the field experiments showed the opposite effect.^39^ It was because of this difference in results, evolutionary biologists came to understand that high predation risk leads to a reduction in immune system investment, which has a different effect on the onset of senescence in a parasite‐free lab environment than in the field.^39^ This insight was only obtained by studying the same species in the lab as well as in the wild.

Similar insights could be obtained in the field of primate cognition. For example, studies on tool use can reach contrasting conclusions when the behavior of captive and wild animals from the same species is compared. For example, bonobos (*Pan paniscus*), who (so far) have not been observed to use tools in the wild to obtain food, were observed to use tools in captivity.^43^ This difference in behavior helps us to obtain insight into the potential variables that play a role in the use of cognitive abilities needed to perform complex forms of tool use. Variables so far identified are: (a) time available for exploration of objects, (b) frequency of access to objects, and (c) levels of distraction (by predation risk or a need to search for food^44^, ^45^). Acknowledging these variables and linking them to ecological variables, such as food availability, provides useful guidelines when designing statistical models that investigate why some natural populations of primates use tools in some habitats, but not in other habitats.^46^, ^47^, ^48^

Unfortunately, the number of cognitive abilities that have been studied in the same primate species, both in the field and the lab, can often be counted on two hands. For example, regarding studies that investigated whether chimpanzees are able to plan for the future, defined as acting for a future motivational state,^22^ I counted seven studies from captivity (observational^49^, ^50^: 2; experimental^51^, ^52^, ^53^, ^54^, ^55^: 5), and only one from the wild (observational: 1,^19^ experimental: 0). For episodic‐like memory, defined as an ability to recall “what.” “where,” and “when” events occurred,^56^ I counted only two captive‐based studies^14^, ^57^ (observational: 0, experimental: 2) and none in wild chimpanzees. The only study of episodic‐like memory in wild primates was done on capuchin monkeys using an experimental approach.^58^

To encourage future comparisons and collaborations between captive and field‐based primatologists, I here apply the expression “unknown, unloved”. As a field‐based observational scientist, in this paper, I explain the advantages of the observational field‐based approach through examples of my own work. By doing so, I will describe some of the advantages of field‐based science as well as the challenges faced in captive‐based science. Yet my aim is not to devalue captive‐based nor experimental research, or to pit us against each other. Rather, my aim is to make captive‐based and experimental scientists think critically about the challenges of their approach and hopefully become more open to, or familiar with, the potential and advantages of observational fieldwork (Box 2). The ultimate aim of this paper is to achieve a better appreciation of the value and urgency of observational field‐based science and to encourage collaboration among scientists using different approaches—enabling us to benefit from our distinct expertise.

## 4 THE IMPORTANCE AND URGENCY OF FIELD STUDIES

### 4.1 Obtaining insight into evolutionary function

Two sources of information are required to study the origin and evolutionary function of a cognitive ability. First, one needs knowledge of how species' cognitive abilities compare. This information has been gathered in a plethora of studies in comparative psychology.^3^, ^8^, ^9^, ^24^, ^34^, ^59^, ^60^ Here, field‐based and captive‐based scientists can reach similar conclusions.^8^, ^61^, ^62^ Second, one needs knowledge on the socio‐ecological context in which species use particular cognitive abilities, to subsequently compare the existence of such contexts across species. Then, both types of knowledge can be used in phylogenetic analyses to test hypotheses about which evolutionary pressures contributed to the positive selection of a cognitive ability.^3^, ^4^, ^7^, ^60^ Hence, it is not sufficient to only compare which animals use particular cognitive skills, but it is also of the utmost importance to compare the conditions in which these animals employ these skills.

Shettleworth^8^ defined cognition as the mechanisms by which animals acquire, process, store, and act on information from the environment, making the understanding of an animal's environment and its interaction with it crucial for understanding its cognition. This environment can be created and controlled in a lab or zoo, though the field allows for understanding how different mechanisms and environmental factors interact and integrate,^8^ and in what contexts cognitive mechanisms are employed and can lead to evolutionary benefits.

### 4.2 External validity

One other advantage of field‐based science is that it provides external validity, meaning that it enables us to test whether cognitive abilities identified in captive settings are used by the animals under natural conditions.^41^, ^42^ Such validity not only increases our confidence that mechanisms were successfully identified,^41^, ^42^ but it also helps us to understand why animals in captive setting sometimes perform poorly in cognitive tasks. One classic example of experiments that lack external validity and result in surprisingly poor cognitive performances can be found in the field of spatial cognition (discussed in 63). In a variety of delayed‐matching‐to‐sample tasks, primates need to remember the spatially distributed objects or food dispensers from which they received food and which they did not. Single locations can be remembered very well when the intervals between exposure and memory testing (retention interval) are as short as 2 min.^64^ However, larger numbers of locations appeared to pose a problem. Initial findings suggested that monkeys are only able to remember a very small number of spatial locations for short time durations.^63^, ^64^, ^65^, ^66^ Some of these studies led to the conclusion that remembering large numbers may be a unique trait in apes^67^ or that memory skills are better in particular species of lemurs compared with others.^68^ In many of these experiments, food locations were not stable, the primates only had one exposure and the retention intervals were often very short and did not match with variables impacting foraging decisions in the wild.^63^, ^65^, ^67^, ^68^ In the wild, visits to novel food sources such as newly emerged fruit are usually separated by 1 day to 1 week.^62^, ^69^, ^70^ Furthermore, only a few food sources have not been visited before.^62^, ^69^, ^70^ For example, in chimpanzees, the average number of fruit trees fed in per day that was “new” within our long consecutive follows of 28–44 days was only four,^71^ and across years our follows suggested that many locations had already been learned in previous years.^69^ When Menzel and Junco^72^, ^73^ tested Andean saddleback tamarins (*Saguinus fuscicollis illigeri*, which is now referred to as *Leontocebus illligeri*), the researchers were the first to use learning schedules that were similar to those likely used in the wild. They introduced novel food locations one at a time with 24 hours between each novel presentation and the testing phase. This approach resulted in (a) one‐trial learning, (b) a memory of up to 30 locations, and (c) food locations being remembered for up to 77 days.^72^, ^73^ These results strongly contrasted to earlier findings that lacked external validity.^66^ Matching the value of other variables that impact foraging decisions in the wild, such as social variables, that is, allowing primates to forage in a social group, likely improved memory performances as well. The emission of food calls, in such social groups are likely associated with positive emotions,^17^, ^74^ which potentially contributed to the consolidation of memory traces. Overall these considerations of natural foraging behavior contributed to the exceptional performances of the tamarins tested^72^, ^73^ and may explain differences in performance in other species.^75^, ^76^

### 4.3 Motivation and challenging complexity

Rosati and colleagues^25^ compared the cognitive performance of chimpanzees (*Pan troglodytes*) and humans (*Homo sapiens*) by offering them a choice between a small immediate reward and a large delayed reward.^25^ Humans surprisingly chose a larger reward, with a delay of 2 min, only 20% of the time that a choice was offered, while chimpanzees did so 70% of the time.^25^ We know, however, that humans are able to delay gratification and can wait for larger rewards, and for example invest money to gain profits years later. Indeed, when the researchers conducted an additional study, and changed the reward to offer money instead of food, humans were more often willing to wait for a larger reward than for a smaller immediate reward. The study is a perfect illustration of how important motivation is when testing cognitive abilities. Currently, a growing number of studies suggest that a lack of evidence for cognitive skills could have been a result of a lack of motivation or interest by the study subject.^77^, ^78^, ^79^ For example, studies that took into account bond strength in subject dyads, before subjects were set up to participate in a cooperative task, appear to be more likely to find evidence for cooperative abilities compared with those studies that paired subjects up randomly.^15^, ^30^, ^80^, ^81^ Cognitive tasks with human demonstrators were completed more successfully by enculturated or human‐oriented apes, which had more contact with (and perhaps more control over) human actions, compared with zoo‐housed apes.^35^, ^77^, ^79^, ^82^ Similarly, chimpanzees who watched a chimpanzee demonstrator performed better in imitation tasks than chimpanzees who watched a human demonstrator.^26^, ^79^ Scientists who conducted the study suggested that the chimpanzees might lack the motivation to imitate another species.^79^ Cognitive experiments that involved researchers dressed up as their study subject's species and behaving like them suggested that the subjects were motivated to look at what the (dressed up) researchers were doing and what they had “in mind”.^27^ This study by Krupenye and colleagues^27^ was the first in decades to find strong evidence that nonhuman apes have a theory of mind.

Other studies indicated how important it is to challenge study subjects and to provide many options when trying to test for cognitive skills.^83^, ^84^ When Schubiger et al.^84^ provided common marmosets (*Callithrix jacchus*) and squirrel monkeys (*Saimiri sciureus*) with the option to indicate where food was hidden in a two‐choice task, for which the chance of success is 50%, both species performed dramatically worse than when they were challenged to remember one location out of nine.^84^ Similarly, when Girndt et al.^83^ found that when apes were offered a choice between pulling two prepositioned rakes to obtain food, where one of the rakes would push the food into a trap, they failed to choose the correct rake above chance.^83^ However, when they were challenged to use only one rake where they had to choose to move the rake to either side (the side with the trap or the side without) to eventually pull the food toward them, 80% of the apes made the correct choice in the first trial. In short, these studies show how complexity in study design can drastically alter results and how more complex or challenging tasks can trigger animals to perform better.

One clear advantage of testing cognitive skills of animals in their natural habitat compared with those in captive settings is that wild animals need to be motivated and interested to perform cognitive skills to obtain naturally occurring food and mates. This does not mean that animals in the wild are always more motivated to employ a cognitive skill than animals in captivity. For example, the motivation of wild animals to participate in field experiments is described as being lower than in captive animals that are likely to have fewer distractions, predetermined foraging plans, or fear of novel objects or food.^21^, ^28^ In addition, there are observational field studies that suggest that motivation (e.g., to walk straight and fast toward sleeping or feeding sites) was low at particular times and areas.^85^, ^86^ Furthermore, not all cognitive skills may necessarily lead to increased access to food or mates. Yet motivation to employ cognitive skills to obtain food (which is the most common reward for cognitive tasks in captivity) is likely to be overall lower in captive than in wild animals for the simple reason that wild animals are not provisioned. Motivation in wild animals may be particularly high in food‐scarce periods, when foragers experience periods in which they catabolize major amounts of body fat, lack particular nutritional compounds, and need to decrease group size.^87^, ^88^, ^89^

### 4.4 Lots of space: Body movement, experience, and cognitive development

The natural environment is characterized by its information complexity and a relatively large‐scale distribution of food and mates. For example, when an animal locates food, it receives sensory information about odor, the sound of other foragers, and visual aspects of food sources. When it has a memory of the food location and value, this knowledge needs to be integrated with sometimes conflicting sensory information.^90^, ^91^ Exposure to a variety of information sources may lead to particular ontogenetic changes in the nervous system.^92^ For example, enabling the development of particular types of mental maps.^10^ In cognitive science, there is a growing consensus that sensory changes produced by motor actions are critical for both development and maintenance of cognitive capacities.^93^ Animals that are never exposed to a large variety of information and that lack the ability for large scale self‐movement to integrate environmental cues may show relatively lower performance levels than animals that have those opportunities.^10^ This effect can be observed in captive‐bred golden lion tamarins (*Leontopithecus rosalia*) that showed low spatial performance when they were released in a large‐scale space^94^ compared with related tamarin species in the wild.^95^, ^96^

While group sizes in a captive environment increasingly approach natural values, group sizes are still lower than those observed in natural habitats in many captive settings.^108^ In addition, the total number of individuals that captive animals have had opportunities to learn from in a life time are relatively low, due to lower rates of dispersion, migration and births. Therefore, animals in the wild have the potential to learn social and ecological skills from a potentially larger number of individuals.^97^, ^98^, ^99^ Individuals do not need to rely on a small number of group members that share their enclosure, especially when these other individuals may all not possess the cognitive capacity at stake. Hence, one of the advantages of working with wild animals is that cognitive abilities have likely developed to their full extent, due to a particularly high variety of social and sensory input and large‐scale movement abilities.

Moreover, wild animals are less likely to endure uncontrollable stress that is known to result in aberrant behaviors and signs of depression in many captive animals.^100^, ^101^, ^102^, ^103^, ^104^ Enrichment conditions have improved substantially over the years, and most experiments are conducted on a voluntary basis. Yet animals that are most often subject to cognitive tests, such as primates, cetaceans, corvids, and elephants are all long‐lived animals,^105^, ^106^, ^107^, ^108^ and a history of uncontrollable stress, including social and nutritional stress and unnatural rearing conditions (e.g., not being reared by the mother) can have long‐term effects on brain morphology.^109^, ^110^ The increased number of studies conducted with sanctuary animals, especially on social cognition is particularly worrisome,^111^ as these animals have likely endured high levels of social and nutritional stress before reaching these sanctuaries^112^ (but see 101). For example, social deprivation during infancy is known to have negative effects on the development of social skills and cognition,^113^ resulting in shorter play bouts that lead to more aggression in chimpanzee orphans compared with mother‐reared chimpanzees.^114^

### 4.5 Urgency

Lastly, it must be emphasized that field studies are urgent. We can study animals in the laboratory or zoo for the next 100 years, but we cannot say the same for many animals in the wild. Natural habitat, especially of tropical forest primates, is disappearing at rapid speed.^115^, ^116^, ^117^ This rapid decline of the rainforest environment and the primate populations that are dependent on it creates a high level of urgency to study animals in their natural habitat.

When collecting behavioral data on wild animals, advanced technologies that enable camera trap or audio triangulation methods are increasingly applied.^99^, ^118^, ^119^, ^120^ Such technological advances enable us to study behavior (through observations or experiments) in a highly noninvasive manner and to avoid the risks associated with habituation, such as disease transmission.^121^ Such approaches make it possible to study wild animals without the need for long‐term commitment to protecting the habituated animals from poachers. It is, however, the long‐term commitments for studying wild populations that stanches their rapid decline, as sheer researcher presence significantly decreases poaching and logging activities in the study areas.^122^ Field primatologists have a tradition of studying a diverse array of primate species^21^, ^108^ including many populations within these species.^123^, ^124^ The number of species and populations clearly outnumber those in captivity,^108^ creating an inspiring potential for comparative research that is disappearing in front of our eyes.^115^, ^116^, ^117^

**Figure 1:**
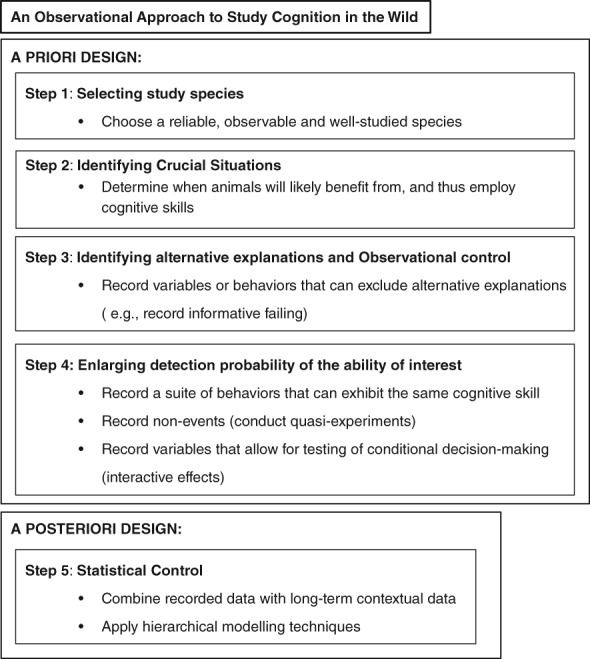
Diagram illustrating the different steps that can be taken to study animal cognition in the wild using an observational approach

Having summarized the advantages of field‐based studies, the question still remains whether it is actually possible to study cognitive abilities in the wild and how we can control for confounding variables, especially when we work with highly endangered animals for which experiments are rarely possible. Tomasello & Call^31^ were not the only ones to express concern about the difficulties of studying animals in the wild. For example, Pritchard et al.^28^ wrote: “As nearly all of this control is difficult if not impossible to achieve in the experimental study of animals cognition in the wild, this can be a major downside to attempting to investigate animal cognition in the wild”. In addition, MacDonald & Ritvo^120^ wrote: “More importantly, obtaining sufficient control over extraneous variables is often impossible.” In the following sections, I describe the approaches I used to deal with many of these proposed difficulties. In addition, I provide guidelines (Figure 1) for future data collection designs.

## 5 FIVE STEPS TO INVESTIGATE COGNITIVE ABILITIES IN WILD ANIMALS BY OBSERVATION

In his seminal work on the aims and methods of ethology, Tinbergen^125^ expresses his concern about the unequal ratio of experimental and observational studies, describing contempt for simple observation as “a lethal trait in any science”. In the same paper, he writes “our science will always need naturalists and observers as well as experimenters; we must, by a balanced development of our science, make sure that we attract the greatest possible variety of talent, and certainly not discourage the man with a gift for observation”. It is, therefore, striking that an updated guideline for observational fieldwork to study animal cognition is lacking to date, despite the many guidelines that are provided for experimental fieldwork.^9^, ^28^, ^29^, ^31^, ^41^, ^42^ Responding to this, as well as to Tinbergen's plea for a more balanced approach (Box 2), I will focus on describing five steps that combine novel and traditional methods.

### 5.1 Step 1: Choosing a study species

The first step in starting an observational field study on animal cognition is to choose the study species. The choice obviously firstly depends on one's questions. However, practical guidelines can be provided (see also Martin & Bateson^126^). Two important criteria proposed by Pritchard et al.^28^ are that the species should be “reliable” and “observable”. Chimpanzees fit these criteria exceptionally well. First, most primates show high levels of site fidelity and can, therefore, be easily relocated across field seasons.^32^ Second, chimpanzees are observable, meaning they do not fly away, dive underwater or live underground but can relatively easily be observed throughout the day. Furthermore, individual chimpanzees, as in most primate species, can be identified without being marked. Another important criterion when choosing a study species or population is that sufficient existing knowledge is available about the behavior and the socio‐ecological environment of the selected animals. The latter is essential for the identification of crucial situations (Step 2) and the exclusion of alternative variables (Step 3).

### 5.2 Step 2: Identifying crucial situations

To identify crucial situations in which animals would likely employ particular cognitive skills, we can make use of the decades of field research on a large variety of species that reveal insights into the challenges animals face in comparison to others in their natural habitat.^127^ For example, previous research shows that chimpanzees have a relatively costly form of long‐distance terrestrial locomotion compared with quadrupedal monkeys,^128^, ^129^ and are morphologically and/or physiologically limited in their digestion abilities.^127^ They cannot eat highly toxic seeds (e.g., *Anthonota fragans*) or mature leaves, as can other primates such as sooty mangabeys (*Cercocebus atys* 18) and many Colobinae.^106^ Yet their large body and brain rely on energy‐rich tropical forest food, such as large crops of ripe fruit.^129^, ^130^ Taï chimpanzees spend 85% of their feeding time on ripe fruit,^74^ and even in fruit scarce periods, females still continue to eat ripe fruit 67% of their time.^131^ Yet ripe fruits are rare; in some chimpanzee territories, ripe fruit‐bearing tree density of edible species was estimated to be 17 times lower than that of trees that bear unripe fruits.^127^ Large ripe fruit crops that can “host” an average chimpanzee party are even rarer and can have a complex distribution in space and time.^127^ To deal with this challenge, we can hypothesize that wild chimpanzees create a mental representation of food locations and values in time, through a large variety of cognitive mechanisms, such as a memory of distant past events, flexible planning and keeping track of proportions of fruit‐bearing trees within species, that is, intuitive statistics.^127^ Hence, by investigating the behavior of chimpanzees during their daily search for ripe fruit we can expect to be able to identify the use of a number of cognitive skills. I provide a detailed example of one more specific foraging situation below.

Identifying situations in which animals likely employ certain behaviors in their natural habitat is nothing new and has a long tradition in the field of ethology.^8^, ^38^ Many studies that revealed that animals use cognitive abilities resulted from considerations of the benefits of using them in the natural habitat.^56^, ^132^ Making a priori predictions about the particular information and skills animals in the wild “should” use is, however, not always straightforward and requires extensive knowledge of their behavior as well as the characteristics of their socio‐ecological environment. For example, rufous hummingbirds (*Selasphorus rufus*) choose flowers in the “correct” spatial location (where they previously found food) over flowers of the “correct” color (at which they previously found food).^28^, ^133^ Considering that hummingbird‐pollinated flowers have evolved in response to hummingbird foraging, it could be expected that they would pay more attention to color.^28^ However, if one considers that flowers become depleted or differ in the amount of food they produce, a prediction that the birds remember the location of the flowers instead of only the color may better match observational findings.^28^ Dependent on the situation (e.g., the spatial scale), animals should prioritize knowledge based on memory over particular forms of sensory information, or the other way around.^28^, ^92^, ^134^

#### 5.2.1 A detailed example: Flexible planning‐ returning to fruit trees at the right time

To identify a situation in which chimpanzees might use flexible route planning, I made use of the following ecological and behavioral information. Rainforests are typically characterized by a large biomass of fruit‐consuming foragers that compete for fruit and can easily deplete a large, productive, ripe fruit‐bearing tree after it has been fed in by a chimpanzee.^135^ Sympatric monkeys, though seldom ripe fruit specialists, do eat ripe fruit and can easily deplete the few ripe fruits that are in a tree when chimpanzees are foraging elsewhere, especially when these fruits are eaten by many other foragers. Figs (*Ficus spp*.), for example, are eaten by more animal species than any other plant genus.^136^ When we visited chimpanzee feeding trees, we found that sympatric species of monkeys, hornbills and squirrels were more likely to be found foraging in a fig tree than in chimpanzee feeding trees of other fruits species.^19^

Small fruits are also a sought‐after resource. They can be eaten by a large number of bird species, for example, because they are simply easier to swallow and can be eaten at faster rates when processing surfaces (e.g., teeth) are small.^137^, ^138^ Long‐term phenology data of chimpanzee feeding trees (11 years) further indicated that ripe fig fruits and small fruits are less persistent.^19^ These fruits are more ephemeral and stay in the trees for shorter periods than other fruits.

The combination of this ecological and behavioral information helped to identify a situation in which it could be beneficial to plan a return to fruit trees and to arrive earlier than competitors at these types of fruits. First, the significant differences in ephemerality level of chimpanzee food sources created a situation in which some of the first food they eat in the morning (hereafter: breakfast food) would be more quickly depleted than others. Second, the variation in distances between chimpanzee sleeping and breakfast sites created a situation in which arrival times would be later at sites that are further away, and would thus likely result in ending travel at a depleted tree if one would not plan to depart earlier to reach such trees. Hence, the combination led to the prediction that chimpanzees would benefit from flexibly planning their early morning departure times (see Step 4; “Question 3: Under what particular conditions do chimpanzees plan?” for a description of how this was tested).

In a similar way, variation among food production rates of individual trees^71^, ^127^, ^139^ creates a situation in which it could pay to be able to differentiate between individual food trees and to remember feeding experiences across seasons or years. This discriminative ability would then enable foragers to not approach just any tree at the start of a season but instead to approach particularly those that are likely to bear large amounts of fruits. Hence, I chose the situation where food production rates varied substantially, to investigate whether or not chimpanzees use a memory of distant past events (see Table 1 for more examples of other cognitive abilities).

**Table 1:** Example situations in which one can expect an animal to employ several cognitive mechanisms

Cognitive mechanisms	Potential crucial situations
**Physical cognition**	
Intuitive statistics/categorization	When the proportion of food‐bearing trees differs substantially between species
What where and when memory	When there are differences in ripening or degradation rates of food
Euclidean map use	When having entered areas, where shortcuts between food sources will decrease travel time
Causal understanding/insight	When being young and needing to learn how to reach food by using a tool
**Social cognition**	
Cooperation	When catching a prey on your own is too difficult
Intentionality/information sharing	When having seen a predator and others, who are related to you, have not
Theory of mind	When wanting to get food that others want as well
Social learning	When having migrated to a new group and need to know who has the highest rank

### 5.3 Step 3: Excluding as many alternative explanations as possible

There are many variables that can explain a behavior. The cognitive mechanism of interest to a researcher is only one of them.^8^ For example, an animal that travels in a straight line toward a food source may have navigated by using a mental representation of the food (using a particular mental map), but it could at the same time have used sensory cues, such as the fruit's conspicuous color, or a searching rule (“go straight until you bump into a food source”). These possibilities challenge cognitive scientists who want to infer the use of a particular cognitive ability by observing behavior.

Hence, to test for particular cognitive abilities, it helps to think of many alternative explanations, ideally before the start of data collection. While determining alternative explanations, we are greatly aided by the growing number of studies on animal behavior in the wild and historical knowledge from long‐term field sites about individuals and their socio‐ecological environment.^21^, ^127^, ^140^, ^141^ This development results in a growing biological knowledge that can and should be used in data collection designs. For example, when designing a statistical model to test for planning abilities by predicting nest departure time, I could make use of a total of 46 field studies ranging from 1960 to 2013 to understand which variables should be included to predict primate sleeping site departure time. In addition, advances in data collection technologies such as high resolution, long‐term bio‐logging^142^, ^143^, ^144^ camera trapping,^98^, ^119^, ^145^ satellite and aerial imaging,^146^ and long‐term field sites^21^, ^140^, ^141^, ^147^ that have decades of contextual data to draw from, provide the data that can help to rule out alternative explanations through statistical methods (Step 5).

Another way to rule out alternative explanations is through observational control. This control is achieved by quantifying the information animals could use, such as the sensory cues that food or mates emit (see an example below). We can also pose the question, what do animals fail to do? (see Question 1 below). These lines of complementary scientific progress allow us to reason more wisely about the variables that most likely affect an animal's behavior.

#### 5.3.1 An example—Are primates using sensory cues or memory?

One of the most difficult challenges facing field‐based scientists who investigate spatio‐temporal memory or route planning is to rule out the use of sensory cues as an alternative explanation for observed behavior. For example, some plant species that rely on seed dispersal can substantially increase the amount of scent emitted from ripe fruit, such that primates can distinguish them more easily from unripe fruits,^148^ and likely detect them from larger distances. One of the most frequently used methods in primatology to rule out the use of sensory cues is to estimate the distances at which the study species can perceive food or other animals.^36^, ^69^, ^85^, ^118^, ^149^ Being primates ourselves sometimes helps to make these distances more realistic. On an olfactory level, humans, similar to nonhuman primates are sensitive to isoamyl acetate, the major component in a large variety of fruit odours.^71^, ^150^, ^151^ Although, the exact link between olfactory receptor genes and odorous ligands is still unclear, humans also have a comparable and even slightly larger estimated number of functional olfactory receptor genes than other primates.^152^ On a visual level, comparative studies indicate that visual acuity thresholds are lower for human than for nonhuman primates, as humans have typically larger eyes and hence larger retinal image size.^153^, ^154^ This enables us to assume that if humans cannot see something, neither can most other diurnal primates. Of course, the human observer's senses may be adapted to different light levels and may not be as trained as those of the study subjects, yet detection distance estimations could make certain simulated detection distances (e.g., >100 m) highly unlikely, leaving the use of spatial memory as the most likely explanation.^155^

Perhaps a better option to rule out the use of sensory cues as an alternative explanation is to incorporate certain behavioral processes, such as feeding competition, to one's predictions. An example of such an approach is the study of Tujague & Janson,^36^ who investigated the approach speed of tufted capuchin monkeys (*Sapajus nigritus*) toward food trees. They ingeniously predicted that the number of individuals that can benefit from early arrival at food trees would increase initially with fruit amount, but would eventually plateau or even decline as food availability becomes large enough to allow all group members to feed. Their data supported the idea that the monkeys were considering the amount of fruit as well as the level of competition they would face at the feeding tree. The tested effect made it highly unlikely that the monkeys had simply been guided by sensory cues, as this would have predicted a linear effect of fruit amount on speed, as opposed to the nonlinear relationship they found.

The easiest observational way to rule out search strategies that are guided by sensory cues is to investigate only approach behavior toward foraging goals that do not emit a strong smell or have a conspicuous color, such as water holes,^144^, ^156^ or to focus on food species or types that do not emit any smell or visual cue that indicates edibility.^18^, ^36^ Another option is to investigate what animals fail to find because food sources are depleted or did not yet produce food. Asking this question can be particularly informative when investigating cognitive mechanisms that can help animals to find food or mates.

#### 5.3.2 Question 1: What do chimpanzees fail to find?—Informative failing

**Figure 2:**
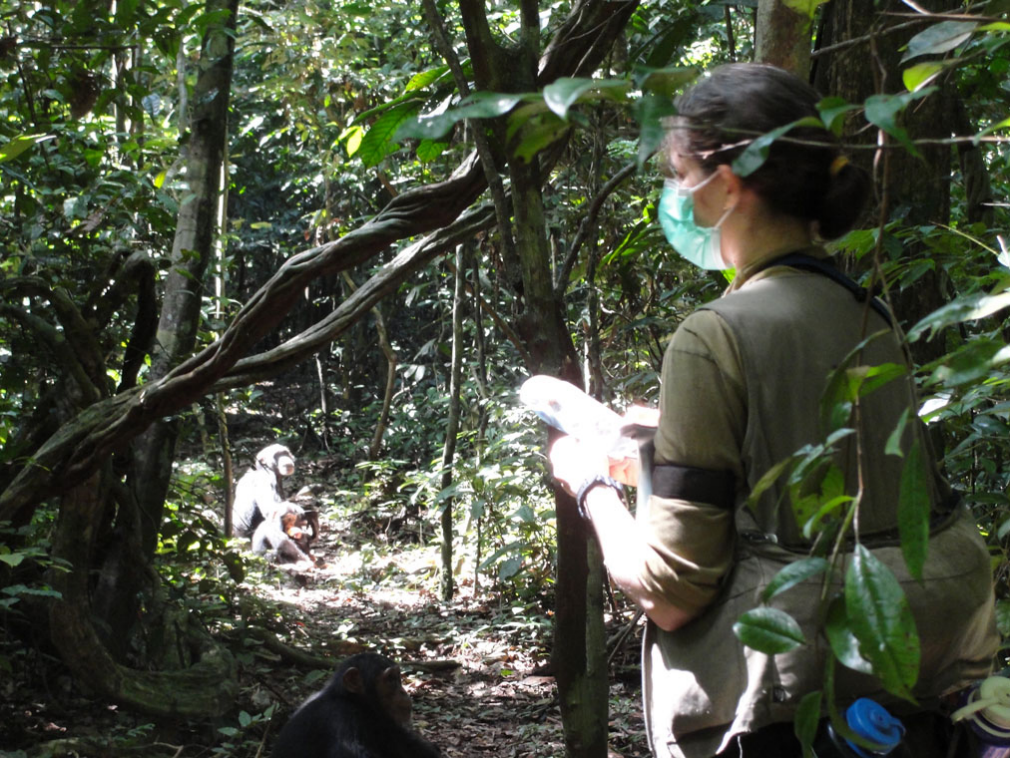
The author collecting data on chimpanzee behavior using a voice recorder and GPS [Color figure can be viewed at http://wileyonlinelibrary.com]

To test whether chimpanzees employ intuitive statistics to improve foraging efficiency, our team followed five female chimpanzees in the Taï National Park in Cote d'Ivoire, totaling 275 days in three food‐scarce periods (Figure 2). During these periods we, marked all trees that the chimpanzees fed in or inspected and recorded their location with a GPS. To know the history of tree visits and to be able to detect the beginning of fruit‐feeding periods, we decided to prioritize on the duration of our follows instead of the number of individuals we followed.^71^ The expectation was that the chimpanzees would use intuitive statistics to more often inspect highly synchronous fruit species, for which they had a high success rate of finding fruits. Hence, we predicted that their inspection behavior would be guided by botanical knowledge. Importantly, we focused on inspections of *empty trees*. These trees did not bear any fruits, nor did they have fruits on the ground, and they could not emit any sensory cues like color or smell, or cues emitted by foraging animals that could have triggered the chimpanzees to look up. By recording when the female chimpanzees looked up at the crown and failed to find food, we gained insight into their expectations about finding food. Since fruits, and thus sensory cues, were absent, we argued that their behavior must have been guided by their botanical knowledge, and was thus able to exclude relevant explanatory variables, such as the use of smell or vision, by *observational control*. In other words, by recording failing behavior, it was possible to determine what chimpanzees were likely expecting—making their failures become informative (*informative failing*). Since the number of inspected trees that were empty was substantial (38% of all inspected trees), we could analyze what influenced the probability of inspecting empty trees. In addition, we measured fruiting synchrony levels of the different fruit species from 11 years of phenology data. To rule out the alternative explanation that the chimpanzees were simply conditioned and were more likely to inspect trees that belonged to species at which they fed earlier more often, we included fruit‐bearing tree density as a control in our statistical model. This also enabled us to control for the possibility that the chimpanzees were sensitive to the absolute number of fruit‐bearing trees they had encountered, regardless of their proportion. Controlling for this, we found that it was the synchrony level and thus the proportion of trees that bore fruits that had a significant effect on inspection probability of empty trees.^71^ Hence by recording informative failing behavior, we found evidence that chimpanzees used intuitive statistics; that is, they had expectations about the different success rates of food finding of particular species, irrespective of their density. This ability to distinguish between proportions of food items was later tested and confirmed in an independent study in captive chimpanzees,^157^, ^158^ providing an example of how field‐based and captive‐based studies on the same species can complement each other and strengthen the evidence for an animal's cognitive ability.

### 5.4 Step 4: Increasing detection probability of the cognitive abilities of interest

In the same way that behavior can be an expression of many different mechanisms; a cognitive mechanism can express itself through many different behaviors. For example, primates may exhibit their use of a spatio‐temporal memory of a food source by (a) rapid travel,^18^, ^159^ (b) highly linear travel,^86^, ^160^ (c) making significant changes in travel direction,^69^, ^161^, ^162^ (d) changing travel direction at long distances before arrival,^74^ or (e) revisiting after particular intervals.^58^, ^163^ Although such behavioral diversity may appear overwhelming at first, it can also be an advantage that can help fieldworkers gain insight into the decision‐making of the animal and detect the use of certain cognitive abilities by applying the rules of parsimony. Before data collection, it can help to design a protocol that considers a suite of behaviors known to potentially express the cognitive skill of interest. Such a protocol should also record behaviors that indicate what animals do not do, or only do when certain conditions are met. To explain this in more detail, I show two examples from my studies on chimpanzees.

#### 5.4.1 Question 2: What do chimpanzees not do?—Quasi‐experiments

To find out whether chimpanzees employed an across‐seasons or year‐long memory of the fruiting states of individual trees, I investigated the probability that chimpanzees would inspect individual trees that they had fed in during previous years. To control for confounding variables, such as sensory cues, I not only investigated what chimpanzees did, but also what they did not do (when they did not inspect, i.e., the nonevents^21^). In an experimental approach, it is as important to know when the animal reacts as well as when it does not react and to record the nonevents.^21^ Similar to an experiment, I sampled the context prior to the observations in the “testing” phase and investigated whether the context was decisive of whether the study subjects did or did not react (event vs. nonevent). Yet, contrary to traditional experiments, I did not manipulate but rather conducted a so‐called *quasi‐experiment*. This is defined by Janson^21^ as a realm of focused observations taken under conditions that account for variation in one or a few hypothesized causal variables, without any actual manipulation of those variables. Thus, I used data from unique follows of one adult female during the three subsequent years. In the first year, our team followed and marked all the feeding trees visited by the target female during 28 consecutive days. During the second and third years, we followed the same female for eight continuous weeks, which included the same period as the first year to ensure that we would cover the same fruiting seasons. Then I analyzed the female's ranging routes in 2011 in relation to the locations of the feeding trees between 2009 and 2010. Next, I investigated which variables influenced the probability that the chimpanzee female inspected one of these trees on the first approach within the respective fruiting season. I recorded when the female inspected but also when she did not inspect (the nonevent) all the trees that she approached to within the detection distance but did not feed on (i.e., trees that were unlikely to bear edible fruit).

By recording events as well as nonevents, we were able to calculate the inspection probability of trees that were approached the year(s) after. We found that after controlling for confounding variables, both the number of feeding visits (familiarity) in the previous years and the maximum amount of fruits found in the feeding trees in previous years had an effect on inspection probability.^69^ Therefore, we were able to find evidence that this chimpanzee used an across‐seasons memory when deciding which fruit trees to monitor by recording what the study animal did, but by also recording what she did not do. These findings support experimental studies in captivity, which showed that chimpanzees can remember tool locations for at least three years.^59^ In this case, the fieldwork provided ideas about the adaptive value of such a memory of distant past events, as fruit‐bearing trees have fruiting intervals that range from 1 to 16 years.^69^, ^127^

The initial idea for a quasi‐experimental approach was developed during a study on spatial memory in mangabeys (*Lophocebus ugandae*).^18^, ^21^ To determine whether this species uses spatial memory of feeding trees' fruiting states, we presampled the context by traversing a monkey group's home range. We then selected a large number of fruit‐bearing and empty trees from the same species prior to following the monkeys. It was only after this presampling that we recorded which trees the monkeys did and did not visit. As my team followed the group for continuous periods of up to 100 days, we were able to compare the visiting probability of fruit trees that had been depleted by the mangabey group earlier in the observation period with the visiting probability of trees that did not bear fruit yet. Since both tree crowns and fruit fall areas were empty, sensory cues could not explain why depleted trees were avoided, and the best explanation of the observed results was that the monkeys were indeed using a spatial memory of fruiting states.^18^ Both studies on foraging cognition in chimpanzees and mangabeys indicated that recordings of nonevents are equally informative as recording what animals do. A similar approach is widely used in the fields of ecology (resource selection^164^) and epidemiology referred to as case–control studies.^165^

#### 5.4.2 Question 3: Under what particular conditions do chimpanzees plan?

To further increase the probability of detecting the use of cognitive abilities by wild chimpanzees, I continued to test what chimpanzees do only when certain conditions are met. I was inspired by Noser & Byrne,^85^ who found evidence that chacma baboons (*Papio ursinus*) departed their sleeping cliffs earlier in fig season than in periods when they fed on other less sought‐after food. Combining this knowledge with the finding of significant differences in ephemerality levels of chimpanzee food, I investigated whether chimpanzees plan to leave their sleeping nest earlier to feed on highly sought‐after ephemeral fruits than when they feed on other fruits. I predicted that nest departure times would be influenced by a number of variables, including the ephemerality level of the fruits (fruit size and type), the fruit genus (figs or other fruits), and a large number of control variables suggested from earlier studies that affect primate sleeping site departure time.^19^ We found that the chimpanzees departed earlier to feed on figs, but only when the fig trees were far away.^19^ Since arrival time for distant figs was similar to arrival time at nearby figs, we concluded that chimpanzees left their sleeping nest earlier to feed on figs that were far away, to make up for travel time and to arrive at about the same time as when the fig trees were close to their feeding trees. Perhaps more intriguing was the finding that the females sometimes departed as much as 2 hours later when they fed on other kinds of fruits. We concluded that chimpanzees delayed their departure when there was little competition with other species, such as for *Panda oleosa* nuts that can only be opened by chimpanzees through tool use. In this case, female chimpanzees (all with young and vulnerable offspring) reversed their behavior relative to moving toward fig trees, avoiding early‐morning departures when they could not easily reach food by climbing short distances through the canopy but had to travel long distances along the forest floor where leopards are active.^19^

Alternative explanations for a given behavior can always be brought forward a posteriori. For example, one could argue that the chimpanzees that happened to depart early could eat from the fig tree, while the ones that happen to depart later missed out on the figs, and hence had to feed on other foods. To discard such explanations, it is crucial to decide a priori to record a suite of behaviors that can indicate planning behavior. For example, the above explanation can be made highly unlikely if one considers (a) the distances the chimpanzees traveled, (b) their speed of approach, (c) the skittish behavior of the early risers treading along the forest floor in the dark, and (d) the finding that no fig trees were inspected nor entered before feeding on the other fruits (see 19) for a discussion of other alternative explanations). First, having recorded the distances and arrival times, we found that the females arrived at about the same time at the breakfast figs that were far away and those that were nearby, making it unlikely that late departures simply resulted in females missing out on figs and ending up eating another kind of fruits. Second, travel speed data informed us that chimpanzees traveled to fig trees more quickly than toward other breakfast sites, supporting that they planned their trips. Third, the observed skittish behavior of the early risers makes it highly unlikely that females would “happen” to depart early for no reason. Finally, if the chimpanzees simply missed out on finding figs after late departures, we should have observed that they inspected or entered depleted trees before feeding, which was not the case. Arguably, each of the above behaviors could potentially be explained by yet another set of alternative explanations; however, following the rule of parsimony, we concluded that flexible planning is the simplest explanation for this combination of behaviors.

It was especially important to think of potential interactive effects when understanding the chimpanzees' decision‐making and the roles of competition and predation risk. In this case, we tested for an interactive effect between fruit type and distance from the nest to the feeding tree and tested what chimpanzees do (e.g., depart early for figs) when certain conditions (e.g., a long distance) are met. Studies that investigate the interactive effects of ecological variables on animal behavior can infer complex cognitive abilities. Other examples can be found in the tool use context. Wild chimpanzees were observed to be more likely to select heavy tools to crack nuts, yet only when they would crack nuts on the ground, but not when they had to take the tool up into a tree to crack nuts on a branch.^20^ Similarly, the same chimpanzees were more likely to select heavy tools, but not when the tools were far away from the anvil and had to be transported over long distances.^20^

The more dimensions an animal needs to take into account, the more likely those particular combinations have never been encountered before and will therefore be *novel*, especially when competition frequently changes these conditions. Chimpanzees reuse the same tools.^166^ Hence tools are likely to be found at different locations each time a chimpanzee revisits the same cracking site. The same level of complex thinking applies to chimpanzee decisions to depart earlier to feed in a distant fig tree compared with those that are nearby. It is true that we do not know where the chimpanzees had been before we started observing them and thus the early departure for distant figs could have resulted from the chimpanzees having learned associations between the time of day and the distance to certain fig trees and a low or high availability of figs (time–place associations^8^). However, we do know that fig trees get depleted after 1.9 feeding visits on average and that Taï chimpanzees make their nest at different locations 98% of the time. Hence, the opportunity for conditional prior learning is limited, making the use of flexible route planning and conditional decision‐making a more plausible explanation of the observed behavior.^19^ Similar novel situations are likely to occur in conditional decision‐making in the social realm, such as whom to mate with, groom or be social with, as ranks and group compositions continuously change.^167^

Not knowing what the animals have done before observations take place can make it difficult to exclude associative learning explanations in field studies, yet it is important to bear in mind that the same problem applies to captive‐based studies where we rarely know what animals have experienced before their arrival in the zoo or laboratory.^168^ Perhaps one could argue that the higher probability of prior associative learning in wild animals will lead to more false positives (type I errors) in field‐based compared with captive‐based studies. However, cases where cognitive abilities in captivity were only confirmed after many experimental studies,^27^ suggest that captive studies are more prone to false negatives (type II errors). This further stresses the importance of studying the same mechanisms in captivity as well as in the wild.

### 5.5 Step 5: Controlling by statistical design—Controlling the uncontrollable

Finally, we can use advanced statistical methods to investigate the cognitive abilities of wild animals. The latest developments in the field of hierarchical or generalized linear mixed modeling^169^, ^170^, ^171^, ^172^ enable us to use repeated observations conducted on the same individuals. This makes it unnecessary to average or aggregate months of data collected on one individual to one single data point, which has dramatic consequences for sample size, power, and statistical analyses. The ability to use repeated observations of the same individuals has become especially valuable for scientists who investigate an animal's long‐term memory by obtaining a complete picture of the animal's experience over time and by observing one individual for extensive periods. These studies only allow a limited number of study subjects within the duration of most scientific funding periods.

For example, in 2004, my colleague and I collected 18 months of data on seven to eight individual male and female mangabeys, respectively. To analyze the data, we were not able to do much more than simple Mann–Whitney U tests.^173^ Statistical tests that are appropriate for small data sets make it impossible to take more than two predictor variables into account.^174^ Mixed or hierarchical modeling techniques such as generalized linear mixed modeling^169^, ^170^ (SI; Fig. S1) enable behavioral scientists to use more data, and thus to include many categorical as well as quantitative predictor variables (to be tested or controlled for) and their interactive effects to predict behaviors.^169^, ^170^ Hence, these techniques enable us to draw much stronger conclusions using purely observational data on animal behavior than was possible in the past.

We furthermore no longer need to throw away data that were recorded close in space or time to avoid a spatio‐temporal dependency of data points. While the approaches are still under development, there are several ways in which scientists can account for autocorrelation between data points taken at short intervals of time or space,^71^, ^171^ allowing researchers to use most or all of their original data. In short, we can embrace all or most of our data and use it to control for many if not all relevant factors. These statistical models, in addition, provide large flexibility with regard to the response variables with diverse distributions (Table 2 169) and also with regard to unbalanced data collection.

**Table 2:** Examples of generalized linear (mixed) models that can be best applied to different types of observational data

Response type	Model type
Normal (e.g., departure time)	Gaussian
Binary (e.g., approach or no approach)	Logistic
Count (e.g., number of visits)	Poisson or negative binomial
Count with many zero's (e.g., number of visits when visits are rare)	Zero inflated Poisson or negative binomial
Count with upper and lower bound (e.g., number of trials correct out of fixed number of trials)	Logistic (only after translating into proportions by use of R)
Continuous with upper and lower bound (e.g., angle deviation)	Beta

#### 5.5.1 An example controlling for evening travel distance to test for future planning

Since chimpanzees make their sleeping nests at different locations in the forest, I could investigate whether chimpanzees position their nest closer or more *en route* to ephemeral fruits. The difference between evening arrival direction and morning departure direction from chimpanzee nests can be influenced by many variables. For example, when figs are rare, a small difference in degree may be caused by the fact that the chimpanzees were traveling toward the fig tree in the evening, but were unable to reach it before dusk, because the fig tree was far away. This could have resulted in chimpanzees making a nest on the way to the morning feeding tree without the use of future planning skills. Therefore, controlling overall travel distances between the last evening and early morning breakfast locations and the possibility that the nest positioning simply reflected a failed attempt to reach a late night feeding site was crucial. By use of statistical control we found that chimpanzees made their nest more *en route* to fig trees used in the morning as opposed to other morning feeding sites, which provided strong evidence that they were indeed planning for the next day.^19^

## 6 CONCLUSIONS AND FUTURE DIRECTIONS

Being selective in the data we record is something field scientists have been trained to do for decades.^125^, ^175^ The information one could record while studying an animal in its natural habitat is often so overwhelming that choices need to be made about the variables to record to best answer the research questions. During this process, priority is often given to behaviors that the target animal performs, such as the trees it visits, how long it eats, how many other animals are present, or whom it grooms. Recording when it does not perform certain behaviors (e.g., when it does not approach or inspect a tree, pick up a tool or groom an individual, or when it fails to find food or mates) does usually not have obvious value. Furthermore, such recordings can require a time investment; one needs to first sample the context (e.g., mark all the trees with and without fruit in the home range), and this will take away time from the behavioral observations one can do within a limited study duration. By providing examples of studies in which such investments paid off, I hope to have created an understanding that this extra time spent can be worthwhile for future studies.

I hope that such future studies will include collaborations between field‐based and captive‐based scientists. Specifically, where similar questions will be asked for the same species in the laboratory and in the field. I envision a variety of joint goals.

First, captive‐based studies can provide insights into the role of genetic predispositions in the development of capacities, such as episodic‐like memory, by being able to confront the animals with challenges they have never faced in the wild (e.g., presenting ice lollies^14^), while field studies can enable us to investigate the evolutionary value of that same mechanism and enlighten us on the type of predispositions we can expect.^18^, ^58^, ^176^

Second, collaboration can help us to better understand the extent of cognitive plasticity. For this, it is essential to study the capacities of populations that live in different environmental conditions.^177^, ^178^, ^179^ The field offers a wide range of variability.^48^, ^180^ Hence, comparative studies on cognitive performances of wild and captive animals provide a wealth of opportunities to determine which factors are important for the development of particular cognitive skills.

Third, collaboration can improve the rigor of field science and to study animals in conditions where experiments are not feasible. To return to an earlier example of alarm calls, determining whether a monkey reacts to a call or to another sensory signal, such as a caller's body movement, is a challenge when you are unable to do experiments. Having more knowledge on the detection distances of these signals could enable field scientists to exclude visual cues and could enable us to extend the research to species and locations where experiments are not feasible. Studies on such sensory abilities, especially on olfactory detection fields, are surprisingly limited to date (but see 150, 151, 153, 154, 181, 182) and would greatly strengthen the conclusions field‐based science can draw.

Fourth, field scientists can provide ideas for new captive‐based testing contexts, or a way to control for biases in performances. For example, many cooperation studies involve food‐sharing activities,^80^, ^81^ which likely results in an unintended bias for high cooperative performance scores in food‐sharing species. Having detailed knowledge on the behavior of wild animals provides an opportunity to control for levels of food‐sharing behavior in a comparative phylogenetic analysis, as well as ideas for new contexts in which to test for cooperative abilities in captive animals.

Finally, there is a new interdisciplinary field emerging to study the adaptiveness of cognitive abilities.^9^ Cognition clearly is essential for a wide range of behaviors that are needed for survival and reproduction.^8^, ^9^ This raises the question of why there is individual variation and plasticity in cognitive performance. Experiments in the lab suggest that some cognitive traits are heritable, yet only a few studies so far have dared to address the question of the consequences of lower levels of cognitive performance and how cognitive abilities or performances are linked to life‐history traits or fitness.^9^ This challenging question can clearly only be answered by combining our best possible collaborative skills.

I envision collaborations where scientists using both the approaches better familiarize themselves with the values of each other's work. In particular, I hope that improved field‐based approaches produce results that obtain a higher status than is sometimes assigned by captive‐based researchers. I especially hope that the guidelines provided here will trigger young scholars to go to the field and reset the balance between field‐based and captive‐based studies (Box 2). By identifying crucial contexts, collecting data on a suite of behaviors (e.g., recording what animals do not do, or only do when certain conditions are met), controlling interfering variables by conducting observational control (e.g., recording what animals fail to find), and by combining this technique with well‐thought‐out statistical models, based on decades of biological knowledge, we are able to infer conclusions about the cognitive abilities of wild animals. What is important to always remember is that every approach has benefits and challenges. Consequently, using complementary approaches is more likely to yield novel insights in primate cognition and move the field in exciting new directions. Perhaps then, we can even make the “impossible” possible.

## Supporting information


**Appendix S1:** Supporting Information

## Data Availability

Data sharing is not applicable to this article as no new data were created or analyzed in this study.
